# Percutaneous Vertebroplasty for Osteoporotic Vertebral Fracture in a Patient with Sickle Cell Disease

**DOI:** 10.5505/tjh.2012.02360

**Published:** 2012-06-15

**Authors:** Mahmut Yeral, Levent Oğuzkurt, Can Boğa, Hakan Özdoğu

**Affiliations:** 1 Başkent University, School of Medicine, Department of Hematology, Ankara, Turkey; 2 Başkent University, School of Medicine, Department of Radiology, Ankara, Turkey

## TO THE EDITOR

Percutaneous vertebroplasty (PVP) is a procedure in which bone cement is injected into vertebrae under fluoroscopic guidance. It was reported to be effective and safe for the treatment of back pain due to compression fractures of the spine [1,2]. As PVP has been successfully used to treat compression fractures of the spine in patients with malignant and non-malignant hematologic diseases, we thought that it might also be beneficial for patients with sickle cell disease (SCD) that have vertebral compression fractures. 

A 22-year-old female with homozygous SCD was admitted to hospital due to severe waist and back pain that had limited her ambulation for the previous 6 months, and she has been bedridden for a month. Physical examination showed tenderness localized to the back and waist regions. Computed tomography (CT) of the vertebrae showed compression fractures at L3, L4, and L5. The compression fracture at L4 was more severe. 

**Figure 1:**
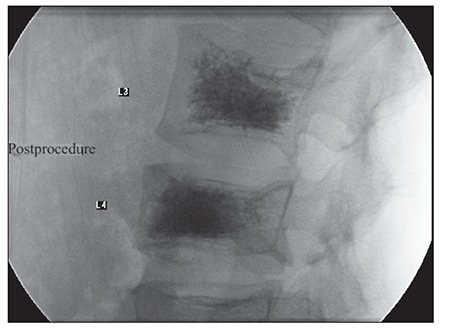
Post-PVP lateral radiogram of the 2 vertebrae shows complete filling of both vertebral bodies with cement. Cement leakage was not observed from the posterior part of either vertebral body.

During the first week of hospitalization, non-steroidal anti-inflammatory drugs (NSAIDs) and opioid analgesics were ineffective for sustained pain relief. PVP was performed on d 8 of hospitalization because her symptoms didn’t improve Under fluoroscopic guidance, an 15-guage needle was advanced to the vertebral body of L4, and then that of L3 via a transpedicular approach. Cement was injected into L3 (3 mL) and L4 (2.5 mL). Cement leakage from the vertebral corpus was not observed, which was confirmed via CT (Figure 1). 

Using a visual analog scale (VAS) we assessed the patient’s functional status and quality of life pre PVP, and 1 week, 1 month, and 6 months post PVP (Table 1) [3]. During the first post-PVP week the patient was able to perform daily activities with only mild pain. No limitations in her movements were observed, but she did occasionally require NSAIDs for pain relief (VAS: 2; ambulation score 2). Pain score was 1 and ambulation score was 2 at 1month and 6 months post PVP. Vertebral fracture in the presented patient negatively affected her quality of life. Due to the risk of thrombosis and acute chest syndrome, the patient had to become ambulatory as soon as possible, thus PVP was performed. The patient experienced pain relief and was ambulatory 24 h post PVP. 

The patient was followed-up closely for possible complications, as to the best of our knowledge she was the first SCD patient to undergo the procedure.The patient had no complications during or after the procedure. 

In conclusion, although more experience is required for more definitive assessment, based on the presented case, PVP seems to be feasible and effective for rapid pain relief and increasing mobility in SCD patients that have vertebral fractures. Written informed consent was optained from the patient to undergo the procedure and for publication of her case. 

**Conflict of Interest Statement **

The authors of this paper have no conflicts of interest, including specific financial interests, relationships, and/ or affiliations relevant to the subject matter or materials included.

## Figures and Tables

**Table 1:**
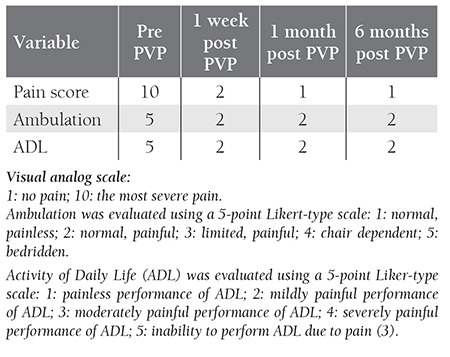
Quality of life quality and functional status pre and post PVP.
